# A docking-based structural analysis of geldanamycin-derived inhibitor binding to human or *Leishmania* Hsp90

**DOI:** 10.1038/s41598-019-51239-0

**Published:** 2019-10-14

**Authors:** Luana Carneiro Palma, Luiz Felipe Gomes Rebello Ferreira, Antonio Luis de Oliveira Almeida Petersen, Beatriz Rocha Simões Dias, Juliana Perrone Bezerra de Menezes, Diogo Rodrigo de Magalhães Moreira, Marcelo Zaldini Hernandes, Patricia Sampaio Tavares Veras

**Affiliations:** 1 Gonçalo Moniz Institute, FIOCRUZ, Laboratory of Parasite – Host Interaction and Epidemiology, Salvador, 40296-710 Brazil; 2 Federal University of Pernambuco, Department of Pharmaceutical Sciences, Recife, 50670-901 Brazil; 3 Gonçalo Moniz Institute, FIOCRUZ, Laboratory of Tissue Engineering and Immunopharmacology, Salvador, 40296-710 Brazil

**Keywords:** Pharmacology, Computational models, Parasitology

## Abstract

Leishmaniasis is a neglected disease that affects millions of individuals around the world. Regardless of clinical form, treatment is based primarily on the use of pentavalent antimonials. However, such treatments are prolonged and present intense side effects, which lead to patient abandonment in many cases. The search for chemotherapeutic alternatives has become a priority. Heat Shock Protein 90 (Hsp90) inhibitors have recently come under investigation due to antiparasitic activity in *Plasmodium* sp., *Trypanosoma* sp. and *Leishmania* sp. Some of these inhibitors, such as geldanamycin and its analogs, 17-AAG and 17-DMAG, bind directly to Hsp90, thereby inhibiting its activity. Previous studies have demonstrated that different parasite species are more susceptible to some of these inhibitors than host cells. We hypothesized that this increased susceptibility may be due to differences in binding of Hsp90 inhibitors to *Leishmania* protein compared to host protein. Based on the results of the *in silico* approach used in the present study, we propose that geldanamycin, 17-AAG and 17-DMAG present an increased tendency to bind to the N-terminal domain of *Leishmania amazonensis* Hsp83 in comparison to human Hsp90. This could be partially explained by differences in intermolecular interactions between each of these inhibitors and Hsp83 or Hsp90. The present findings demonstrate potential for the use of these inhibitors in the context of anti-*Leishmania* therapy.

## Introduction

Leishmaniasis, a disease caused by the protozoan parasite of the *Leishmania* genus, typically manifests, depending on the parasite species, in three main clinical forms: visceral, cutaneous or mucocutaneous^1^. Despite its wide distribution around the world, leishmaniasis is considered a neglected tropical disease that is endemic in 98 countries^2^. Regardless of clinical form, antileishmanial therapy is predominantly based on the use of pentavalent antimonials and amphotericin B. Unfortunately, these drugs present high toxicity and severe side effects, in addition to necessitating extended courses of treatment that often result in abandonment^3,4^. Due to decreased side effects, treatment with liposomal amphotericin B has been adopted in some developed countries, but the high cost of this therapy restricts its usage in large populations^5^. Thus, the search for highly effective and less toxic alternatives has become a priority.

To this end, Heat Shock Protein 90 (Hsp90) inhibitors, classically employed in cancer treatment, have recently come under investigation with respect to leishmaniasis treatment^6,7^. Hsp90, a highly conserved molecular chaperone expressed by eubacteria and all eukaryotes, plays an important role in maintaining cellular homeostasis. In *Leishmania* spp., Hsp90 ortholog is also named as Hsp83. Importantly, this protein participates in the correct folding of its client proteins, such as transcription factors and protein kinases, mainly involved in cell cycle regulation and cell signaling pathways^8^. Structurally, Hsp90 is a homodimer, in which each monomer is formed by three distinct domains: the N-terminal domain (NTD) that contains its ATP binding site, the middle domain, which is the main binding site for its client proteins, as well as some co-chaperones that aid Hsp90 in protein folding, and the C-terminal domain that is the critical site for both client-binding and the dimer formation of Hsp90^9^. During the molecular cycle of Hsp90, the protein undergoes several conformational alterations: after ATP binding to the NTD, Hsp90 reaches the first intermediate state by repositioning a lid segment, which leads to concomitant structural changes that result in the dimerization of the NTDs. These domains then become associated with M-domains, and in its second intermediate state, the chaperone assumes a closed conformation, in which ATP hydrolysis and ADP release occur. Finally, Hsp90 forms a large protein complex together with client proteins and co-chaperones, which play the pivotal role of regulating Hsp90 activity^10^.

The inhibition of Hsp90 results in drastic consequences to eukaryotic cells, as it leads to the formation of poorly-folded proteins that will ultimately be degraded via the proteasome proteolytic pathway, there by interfering with cell growth and survival^11^. Several compounds are known to inhibit Hsp90 activity. These can act directly by affecting Hsp90 binding, or indirectly, through the inhibition of Hsp90 co-chaperones^12^. Geldanamycin (GA), an antibiotic from the benzoquinone ansamycin family, is obtained via fermentation of the bacterium *Streptomyces hygroscopicus*. Chemically, GA is formed by a planar macrocyclic ansa bridge structure linked to a quinone moiety. GA binds directly to Hsp90, more specifically to this protein’s ATP binding site present in the NTD^13–15^. Several GA analogues have been synthesized, including 17-allylamino-17-demethoxy geldanamycin (17-AAG) and 17-dimethylamino ethylamino-17-demethoxygeldanamycin (17-DMAG). Both compounds arise from the inclusion of different amines in the non-essential methoxyl group present on carbon 17 and, similarly to GA, both bind to the ATP binding site in the NTD of Hsp90^16,17^.

Recent studies have demonstrated interference by GA and its analogues on the growth and survival of a variety of protozoan parasites. GA treatment was shown to inhibit the growth of intracellular *Plasmodium falciparum* in infected erythrocytes. Pulldown experiments revealed that GA also binds specifically to *P*. *falciparum* Hsp90 and this inhibitor showed a higher binding ability to parasite Hsp90 than to human Hsp90^18^. Pallavi, *et al*.^18^ further demonstrated that 17-AAG inhibited the growth of *P*. *falciparum* isolated from patients. More recently, Murillo-Solano, *et al*.^19^ demonstrated that GA, 17-AAG and 17-DMAG suppressed the growth of *P*. *falciparum* at concentrations shown to be non-toxic to host cells. In a murine model of trypanosomiasis, *Trypanosoma evansi* growth was inhibited in mice treated with 17-AAG in comparison to control animals that only received the vehicle^18^. In addition, Pizarro *et al*.^20^ demonstrated that a number of ligands, including GA, inhibited the growth of *Trypanosoma brucei* and bound strongly to the Hsp83 of this parasite.

The treatment of *Leishmania donovani* promastigotes by GA was shown to result in the inhibition of parasite growth in a dose-dependent manner^21^. Li *et al*.^22^ also demonstrated that GA induced apoptosis in axenic *L*. *donovani* promastigotes. Moreover, the treatment of axenic *L*. *braziliensis* promastigotes with 17-AAG induced parasite death in a dose-dependent manner. A similar effect was observed in macrophages infected by *L*. *braziliensis*, in which 17-AAG caused a significant reduction in parasite load *in vitro*^7^. Recently, derivatives of reblastatin, a GA-related compound, have been shown to bind to *L*. *braziliensis* Hsp83 and exert inhibitory activity over this protein^23^. Treatment with 17-AAG was further shown to reduce the percentage of macrophages infected by *L*. *amazonensis*, as well as the number of intracellular parasites; these effects occurred at doses that were not even remotely toxic to macrophages. Moreover, while 17-AAG not only killed parasite promastigotes, known to colonize the insect vector, it also neutralized the amastigote forms found within intracellular compartments in host cells. These leishmanicidal effects on intracellular parasites were shown to be irreversible and independent of the production of nitric oxide, superoxide or pro-inflammatory molecules by macrophages^6^.

Taken together, these findings provide convincing evidence that *Leishmania* presents higher susceptibility to Hsp90 inhibitors than host macrophages. Accordingly, we hypothesized that this phenomenon might be due to differences in intermolecular interactions occuring between GA-derived Hsp90 inhibitors and parasite Hsp83, in comparison to host Hsp90.

To test this hypothesis, we initially performed a cell viability assay to evaluate differences in susceptibility to GA-derived Hsp90 inhibitors by comparing *L*. *amazonensis* promastigotes to a human macrophage cell-line. We employed an *in silico* approach via molecular docking to investigate the types of chemical interactions produced during the binding of these inhibitors to the NTD of Hsp90 in parasites and host cells.

Using a computational model, we demonstrated that the GA class of compounds (GA, 17-AAG and 17-DMAG) presents an increased tendency to bind to the NTD of parasite Hsp83 compared to the NTD of human Hsp90, which may, in part, explain the strong and selective antiparasitic activity exhibited by these anti-leishmanials.

## Results

### *L*. *amazonensis* presents greater susceptibility to Hsp90 inhibitors than the differentiated THP-1 cell-line

**Figure 1:**
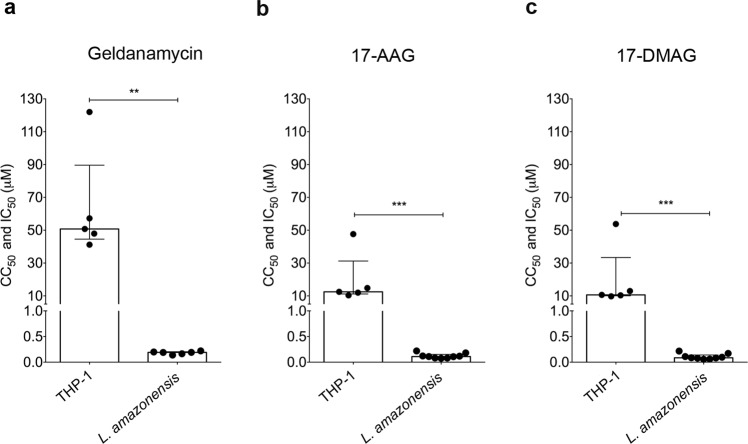
*L*. *amazonensis* presents greater susceptibility to Hsp90 inhibitors than the differentiated THP-1 cell-line. *L*. *amazonensis* axenic promastigotes and differentiated THP-1 cells were treated with (**a**) GA, (**b**) 17-AAG or (**c**) 17-DMAG in a 12-step serial dilution assay. Graphs depict IC_50_ (*L*. *amazonensis*) and CC_50_ (THP-1 cell-line) values for each respective inhibitor. Mann Whitney test ***p = 0.001, **p = 0.0043.

**Figure 2:**
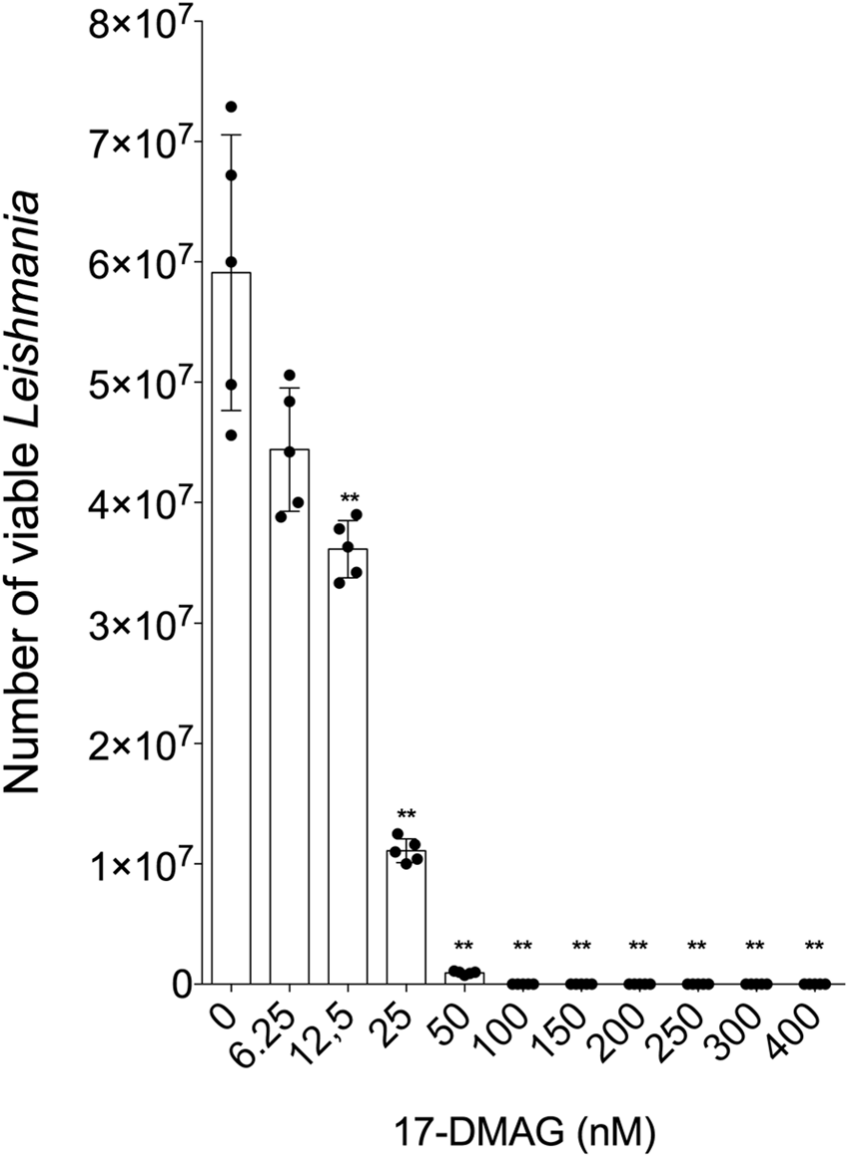
Intracellular viability of *L*. *amazonensis*-infected THP-1 cells treated with 17-DMAG. THP-1 cells (5 × 10^5^ per well) were plated in RPMI medium with PMA (100 nM) to promote differentiation into macrophages, then infected with *L*. *amazonensis* promastigotes (10:1) for 6 h at 35 °C. After washing to remove non-internalized parasites, cells were treated with 17-DMAG at concentrations of 6.25 nM, 12.5 nM, 25 nM, 50 nM, 100 nM, 150 nM, 200 nM, 250 nM, 300 nM and 400 nM (five replicates per concentration) for 72 h at 35 °C. Cells were washed, Schneider’s complete medium was added and, after four days at 24 °C, parasites were counted in a Neubauer chamber. Mann Whitney test **p < 0.01.

Initially, we evaluated differences in parasite and host cell susceptibility to Hsp90 inhibitors. Cell viability assays evaluating the leishmanicidal activity and cytotoxicity (THP-1 cell-line) of GA, 17-AAG and 17-DMAG revealed that GA reduced parasite cellular viability by 50% at a concentration of 0.1910 μM (Q1: 0.16 μM and Q3: 0.20 μM), versus a concentration of 50.77 μM (Q1: 44.57 μM and Q3: 89.62 μM) for the THP-1 cell-line (*p* = 0.0043) (Fig. 1a). This data was used to calculate the selectivity index (SI) of GA at 266, i.e. 266 times more GA is needed to kill 50% of the THP-1 cell-line compared to 50% of the *L*. *amazonensis* promastigotes. 17-AAG was shown to reduce parasite viability by 50% at a concentration of 0.1105 μM (Q1: 0.08 μM and Q3: 0.15 μM), while a significantly higher CC_50_ value of 12.47 μM (Q1: 11.17 μM and Q3: 31.22 μM) was calculated for the THP-1 cell-line (*p* = 0.001) (Fig. 1b), resulting in an SI of 113 for 17-AAG. The IC_50_ value for 17-DMAG was 0.0861 μM (Q1: 0.07 μM and Q3: 0.14 μM) with respect to *L*. *amazonensis* promastigotes as compared to a CC_50_ value of 10.60 μM (Q1:10.04 μM and Q3: 33.39 μM) for the THP-1 cell-line (*p* = 0.001) (Fig. 1c), resulting in a corresponding SI of 123. GA presented the highest SI when compared to 17-AAG or 17-DMAG. Taken together, these results indicate that all the inhibitors evaluated clearly presented much higher toxicity against *L*. *amazonensis* compared to the host cell. We also performed cytotoxicity assays on human lung fibroblasts (MRC-5 cells) treated with 17-AAG or 17-DMAG. We observed that, as in THP-1 cells, the inhibitors tested were shown to be non-toxic, with a CC_50_ of 13.24 μM for 17-DMAG and 86.15 μM for 17-AAG (Supplementary Fig. S1). To evaluate whether the toxicity observed in promastigotes would also be seen in amastigotes, we performed an intracellular viability assay in infected macrophages and treated with 17-DMAG. As in promastigotes, 17-DMAG also presented high toxicity to amastigotes. At concentrations over 100 nM of 17-DMAG, no viable parasites were observed. Interestingly, the IC_50_ value obtained was as low as 13.4 nM (Fig. 2).

### Hsp90 inhibitors present different docking interactions during binding to the NTD of *L*. *amazonensis* Hsp83 than the NTD of human Hsp90

**Figure 3:**
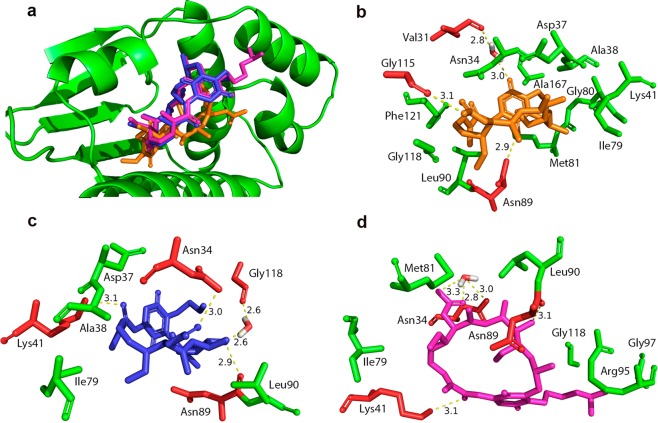
*L*. *amazonensis* Hsp83 binding site with inhibitors and interaction types. (**a**) *L*. *amazonensis* Hsp83 binding site with GA (orange), 17-AAG (blue) and 17-DMAG (magenta). (**b**) GA (orange), (**c**) 17-AAG (blue) and (**d**) 17-DMAG (magenta) and the Hsp83 residues that make hydrophobic contacts (green) and hydrogen bonds (red).

Using an *in silico* approach, it was observed that *L*. *amazonensis* Hsp83 binding site to GA, 17-AAG and 17-DMAG was similar, as expected (Fig. 3a). Considering both the NTD of *L*. *amazonensis* Hsp83 model and the *Homo sapiens* Hsp90 target protein, all three compounds tested in this study formed hydrogen bonds, bridged by water molecules, with amino acid residues at the target binding site.

Taking into consideration the *L*. *amazonensis* Hsp83 model, modeling analysis revealed that GA (Fig. 3b) possibly formed a hydrogen bond, bridged by a water molecule, at a distance (donor-acceptor) of 3.0 Å to residue Val31, hydrogen bonds at a distance of 2.9 Å (Asn89); 3.1 Å (Gly115) and made hydrophobic contacts with residues Asn34, Asp37, Ala38, Lys41, Ile79, Gly80, Met81, Leu90, Gly118, Phe121 and Ala167. The modelling suggests that 17-AAG inhibitor (Fig. 3c) formed a hydrogen bond, bridged by a water molecule, at a distance of 2.6 Å to residue Gly118, hydrogen bonds at a distance of 3.0 Å (Asn34); 3.1 Å (Lys41); 2.9 Å (Asn89) and presented hydrophobic contacts with most of the same residues that made contact with GA, Asp37, Ala38, Ile79 and Leu90. On the other hand, the analysis revealed 17-DMAG (Fig. 3d) probably formed two hydrogen bonds, bridged by a single water molecule, at distances of 3.3 Å and 2.8 Å to residue Asn34, in addition of making hydrogen bonds with the residues Lys41 and Asn89 at a distance of 3.1 Å. Analysis indicates that 17-DMAG also made hydrophobic contacts with the residues Ile79, Met81, Leu90, Arg95, Gly97 and Gly118.

**Figure 4:**
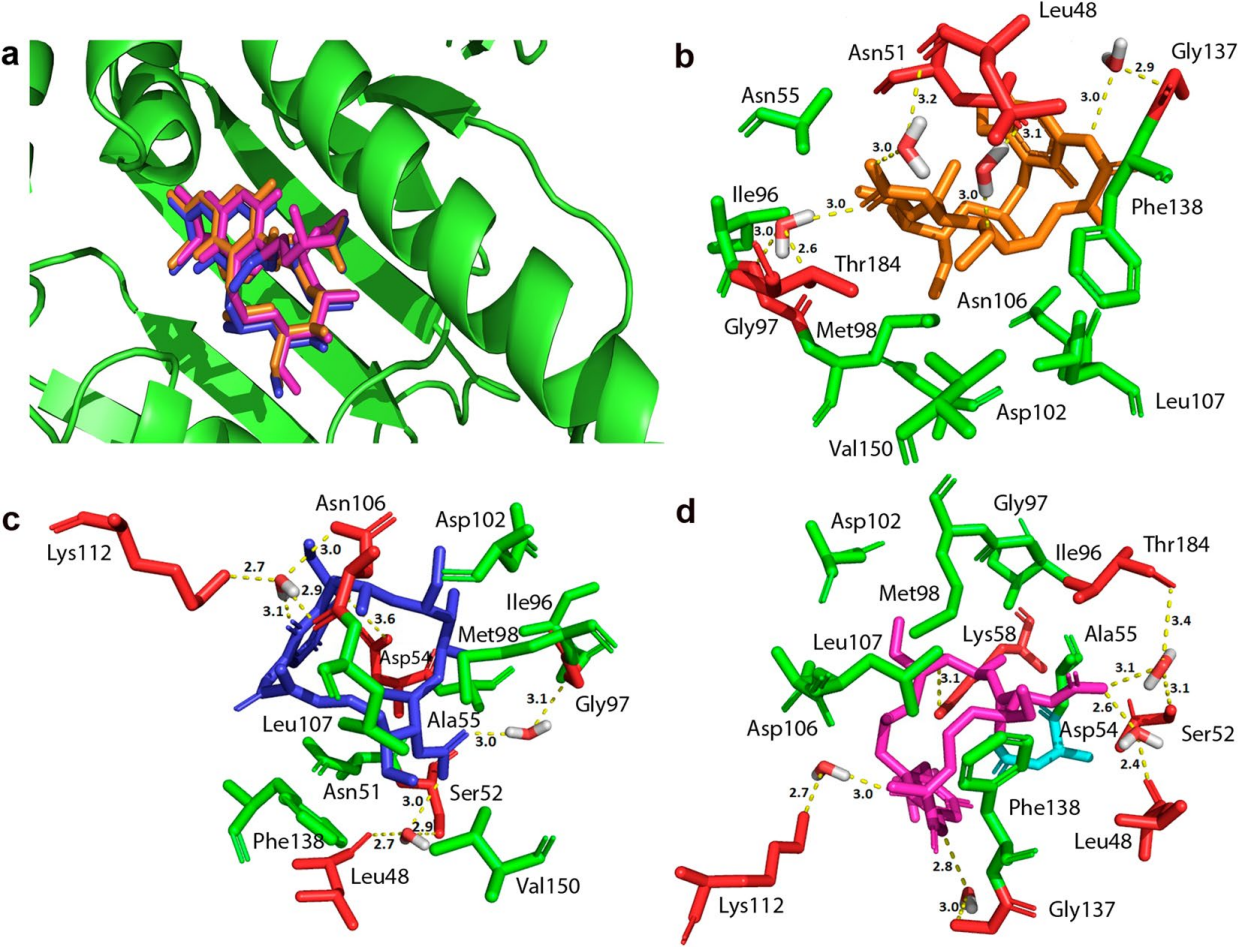
*Homo sapiens* Hsp90 binding site with inhibitors and interaction types. (**a**) *Homo sapiens* Hsp90 binding site with GA (orange), 17-AAG (blue) and 17-DMAG (magenta). (**b**) GA (orange), (**c**) 17-AAG (blue) and (**d**) 17-DMAG (magenta) and the Hsp90 residues that formed hydrophobic contacts (green), salt bridges (cyan) and hydrogen bonds (red).

As expected, we observed that for all tested inhibitors, GA, 17-AAG and 17-DMAG, the binding site of human Hsp90 was identical (Fig. 4a). Modelling analysis also suggests that the natural compound, GA (Fig. 4b), formed hydrogen bonds, bridged by water molecules, with the following amino acid residues at 3.0 Å of distance: Leu48, Asn51, Gly97, Thr184 and Gly137, as well as made hydrophobic contacts with residues Asn55, Ile96, Met98, Asp102, Asn106, Leu107, Phe138 and Val150. In addition, the GA analogue, 17-AAG (Fig. 4c), possibly formed hydrogen bonds, bridged by water molecules, with amino acid residues at the following distances: 3.0 Å (Leu48 and Ser52), 3.6 Å (Asp54), 3.0 Å (Gly97) and 3.1 Å (Asn106 and Lys112). Also, 17-AAG made hydrophobic contacts with most of the same residues as GA: Ala55, Ile96, Met98, Asp102, Leu107, Phe138 and Val150, except for Asn51. On the other hand, 17-DMAG (Fig. 4d) probably formed hydrogen bonds, bridged by water molecules, to other amino acid residues different from GA. The distances between the compound and the amino acids were (donor-acceptor): 2.6 Å (Leu48), 3.1 Å (Ser52 and Thr184), 3.0 Å (Lys112) and 2.8 Å (Gly137). The analysis indicates that Hsp90 inhibitor also formed a hydrogen bond with residue Lys58 at a distance of 3.1 Å, in addition to a salt bridge with residue Asp54 and made hydrophobic contacts with the following residues: Ala55, Ile96, Gly97, Met98, Asp102, Asn106, Leu107 and Phe138.

The Balloon program generated six conformer ensembles of each compound illustrated in Supplementary Material (Supplementary Fig. S2). The ChemPLP analysis scores for all six conformations of each Hsp90 inhibitor are listed in Table 1. This modelling analysis indicates that all the studied inhibitors presented higher scores when docking to the NTD of *L*. *amazonensis* Hsp83 than the NTD of *Homo sapiens* Hsp90: GA (73.89 vs. 54.30), 17-AAG (75.81 vs. 56.02) and 17-DMAG (78.02 vs. 54.53), respectively. Also, with the exception of 17-AAG, which the ring conformation was the same as the co-crystalized ligand former conformation, each ligand’s top ranked conformation for the NTD of *L*. *amazonensis* Hsp83 (3H80.1.A) showed higher ChemPLP score values when compared with the top ranked conformation for the NTD of *Homo sapiens* Hsp90 (1YET).

**Table 1:** ChemPLP scores for each inhibitor and its respective conformations when bound to the NTDs of *L*. *amazonensis* Hsp83 or human Hsp90.

Inhibitor		Conformation 1	Conformation 2	Conformation 3	Conformation 4	Conformation 5	Conformation 6	Average
GA	La Hsp83	73.48	74.37	83.16	72.13	70.51	69.67	73.89
	Human Hsp90	73.38	52.45	48.68	51.51	46.62	53.14	54.30
17-AAG	La Hsp83	65.83	81.82	73.75	79.50	72.50	81.47	75.81
	Human Hsp90	70.05	47.94	46.34	63.80	51.19	56.80	56.02
17-DMAG	La Hsp83	81.79	80.58	85.35	69.12	73.13	78.17	78.02
	Human Hsp90	68.85	51.11	50.94	54.94	52.38	48.97	54.53

## Discussion

The treatment of leishmaniasis continues to represent a substantial challenge, since a range of disadvantages are associated with most available drugs, including severe side effects, prolonged periods of treatment and low adherence to therapy by many patients. As a result, alternative chemotherapies are under investigation in an attempt to mitigate these limitations. The present study evaluated three Hsp90 inhibitors: GA, 17-AAG and 17-DMAG, all compounds with recently recognized anti-parasitic activity. The latter two are currently undergoing clinical trials to evaluate cancer treatment.

The present report investigated differences in the susceptibility of *L*. *amazonensis* promastigotes and differentiated THP-1 cells to this family of Hsp90 inhibitors. Our results show that all tested inhibitors presented greater toxicity to parasites than to host cells, as evidenced by differences in IC_50_ (*Leishmania*) and CC_50_ (THP-1 cell-line) values (Fig. 1). Similarly to the present study, Murillo-Solano, *et al*.^19^ demonstrated that GA, 17-AAG and 17-DMAG were able to inhibit the growth of *P*. *falciparum* at submicromolar concentrations, whereas growth inhibition in human pulmonary fibroblasts was only observed at levels over 10 μM. In addition, *L*. *amazonensis* and *L*. *braziliensis* promastigotes were also found to be more susceptible to treatment with 17-AAG than murine macrophages^6,7^. Moreover, the elevated SI values observed for each inhibitor (GA: 266; 17-AAG: 113; 17-DMAG: 123) further serve to confirm the observed differences in susceptibility between parasites and host cells with regard to treatment involving these compounds. Of the three inhibitors, the highest selectivity index values were obtained from GA, seemingly suggesting that this inhibitor could be an optimal choice for use in future studies. However, since GA treatment has been associated with hepatotoxicity, taken together with compound’s low water solubility and chemical instability, it is considered infeasible as a leishmaniasis treatment^24^. 17-AAG and 17-DMAG, both synthesized from GA, exhibit lower toxicity and, in the case of 17-DMAG, greater solubility in water than GA, which increases the likelihood of use in future studies^25^. In sum, these observations regarding differences in susceptibility between parasites and host cells reinforce the idea that Hsp90 represents a druggable target for the development of novel antileishmanials.

Based on strong evidence demonstrating interactions between the GA family of compounds and Hsp90, we postulated that human and parasitic proteins may present differences upon binding with these compounds.

Using an *in silico* approach, we evaluated interactions between GA, 17-AAG or 17-DMAG, and the NTDs of *L*. *amazonensis* Hsp83 or human Hsp90. With respect to the NTD of *L*. *amazonensis* Hsp83, the analysis suggested that all three inhibitors formed both direct and water-bridged hydrogen bonds and hydrophobic contacts with several residues (Fig. 3). By contrast, all three inhibitors formed numerous hydrogen bonds, most of them being water-bridged, upon interaction with the NTD of human Hsp90, in addition to making hydrophobic contacts with several residues common to each compound (Fig. 4). The water-mediated hydrogen bonds between ligands and the amino acid residues of the NTD of trypanosomatid Hsp83 seems to be important, as it “mimics the position of one of the phosphate groups of ATP”, as seen in a study that analyzed the molecular interactions between compounds and the NTD of *T*. *brucei* Hsp83^20^. Additionally, another study by Nilapwar *et al*. indicated that water-mediated hydrogen bonds, direct bridging or forming a network of water molecules, can reduce “the value of the ΔCp through a net reduction in rotational and vibrational bonding modes”^26^. Furthermore, in according with these authors’ findings, there seems to be commonality with regard to the position of water molecules found at the binding site of crystal structures of the NTD of Hsp90, available at the Protein Data Bank (PDB), which reinforces the importance of these water-mediated hydrogen bonds. The numerous hydrogen bonds water-bridged, observed in interactions between the NTD of human Hsp90 and GA, 17-AAG or 17-DMAG, do not offer a similar stabilizing effect when compared to the hydrogen bonds that formed directly between these compounds and the target’s amino acids residues. For instance, while GA only formed water-bridged hydrogen bonds when interacting with the NTD of human Hsp90 binding site residues, this compound has performed one water-bridged hydrogen bond and two direct hydrogen bonds (not water-bridged) when interacting with the NTD *L*. *amazonensis* Hsp83. Additionally, when taking into account only direct hydrogen bonds interactions, the inhibitors 17-AAG and 17-DMAG have formed a single hydrogen bond non-bridged (each) with the NTD of human Hsp90 binding site, while these compounds performed two (each) non-bridged hydrogen bonds when interacting with the NTD of *L*. *amazonensis* Hsp83 binding site. Moreover, similarities observed in the types of interactions between each of the three inhibitors and the NTDs of *L*. *amazonensis* Hsp83 or human Hsp90 could be explained by the chemical similarity observed between GA and its derivatives, 17-AAG and 17-DMAG^27^.

Regarding the *in silico* structural analysis, based on a docking study of the interaction between these three GA-derived inhibitors and the NTDs of *L*. *amazonensis* Hsp83 or human Hsp90, our results identified that GA, 17-AAG and 17-DMAG may present increased tendency to bind to parasite Hsp83 in comparison to human Hsp90 (Table 1). A previous *in vitro* study by Pizarro *et al*.^20^ involving the compounds GA, 17-AAG and 17-DMAG found that all exhibited stronger binding to the NTD of *T*. *brucei* Hsp83 than to human Hsp90. In addition, crystallographic analysis revealed that other compounds that also bind to the NTD of *T*. *brucei* Hsp83 alter the conformation of this domain, which suggests structural flexibility in the chaperone that may influence selectivity^20^. Another *in vitro* study by Pallavi *et al*.^18^ demonstrated that GA has higher binding ability to *P*. *falciparum* Hsp90 than to human Hsp90. Reinforcing our results, some derivatives of reblastatin, a GA-related compound, were recently shown to exhibit increased binding to *L*. *braziliensis* Hsp83 than to human Hsp90^23^. Similarly, Kanwar *et al*.^28^ demonstrated that SNX-2112 analogs, inhibitors of Hsp90, exhibit leishmanicidal activity but are not cytotoxic to the host. These authors suggest that this may occur due to increased selectivity by the tested analogs for *Leishmania* Hsp83 in comparison to human Hsp90. With respect to the interaction of GA to *L*. *braziliensis* Hsp83, Silva and collaborators reported changes in binding ability when the full-length protein underwent alterations, with increased binding observed when this inhibitor was bound only to the NTD of *L*. *braziliensis* Hsp83^29^. While it is possible that the higher ChemPLP scores observed in the *in silico* docking of GA, 17-AAG and 17-DMAG to the NTD of *L*. *amazonensis* Hsp83 would be also observed *in vitro*, additional studies employing recombinant *L*. *amazonensis* Hsp83 and human Hsp90 must be performed. Finally, these findings may also be related to the nature of intermolecular interactions established between the inhibitors and their respective proteins. The presence of additional direct hydrogen bonds (not water-bridged), which interact with the NTD of *L*. *amazonensis* Hsp83, may offer a greater stabilizing effect than the presence of fewer direct hydrogen bonds, which was seen in binding to the NTD of human Hsp90.

In sum, the results obtained herein are consistent with previous studies demonstrating differences in susceptibility of *Leishmania* parasites and host cells to Hsp90 inhibitors, which was evidenced by higher parasite toxicity. This finding may be justified by the higher ChemPLP scores observed when the inhibitors docked to the NTD of parasite Hsp83. Nonetheless, future *in vitro* studies should be conducted to evaluate the binding of these three inhibitors to recombinant parasite Hsp83 versus human Hsp90. Importantly, our study did not consider the effects of conformational changes in the NTD and/or additional Hsp90 domains caused by the inhibitors, which would likely influence the binding ability of the GA-derived inhibitors to parasite Hsp83 in comparison to human Hsp90.

The present study demonstrated that, in addition to previous reports regarding GA and 17-AAG^6,7^, 17-DMAG also demonstrates substantial anti-leishmanial activity. The findings presented herein may justify the increased tendency of these inhibitors to bind to the NTD of parasite Hsp83 than to that of human Hsp90, and provides support for the further study of this family of Hsp90 inhibitors in the development of novel antileishmanials.

## Methods

### Parasites

Parasites of *L*. *amazonensis* (MHOM/Br88/Ba-125) were obtained from the Laboratory of Host - Parasite Interaction and Epidemiology - Gonçalo Moniz Institute (Bahia-Brazil). Axenic *L*. *amazonensis* promastigotes were grown in Schneider’s Insect Medium (Sigma, St. Louis, MO, USA) supplemented with 50 μg/mL gentamicin (Gibco, Grand Island, NY, USA) and 10% fetal bovine serum (Gibco, Grand Island, NY, USA). Cultures were maintained in an incubator at 24 °C until *in vitro* experiments were performed.

### THP-1 and MRC-5 cell-line

Monocytic human THP-1 cells and MRC-5 cells were grown in Roswell Park Memorial Institute (RPMI) 1640 medium (Gibco, Grand Island, NY, USA) supplemented with 2 g/L sodium bicarbonate (Sigma, St Louis, MO, USA), 25 mM N-2-hydroxyethyl piperazine-N’-2-ethane-sulfonic acid (HEPES)(Sigma, St Louis, MO, USA), 1 mM sodium pyruvate (Sigma, UK), 2 mM glutamine (Gibco, Grand Island, NY, USA), 20 g/mL ciprofloxacin (Isofarma, Precabura, CE, BR) and 10% fetal bovine serum (Gibco, Grand Island, NY, USA). Cultures were kept in an incubator under 5% CO_2_ at 37 °C until the time of compound toxicity assessment.

### Anti-*Leishmania* assay

Axenic *L*. *amazonensis* promastigotes in logarithmic phase were distributed on 96-well plates (4 × 10^5^ parasites/well) in Schneider’s complete medium and treated with GA (Invivogen, San Diego, CA, USA), 17-AAG (LC Laboratories, Woburn, MA, USA) or 17-DMAG (LC Laboratories, Woburn, MA, USA) in 12-step serial dilutions (1:2) at an initial concentration of 2 μM. After 72 h of treatment at 24 °C, parasites were incubated with 10% Alamar Blue^®^ (Invitrogen, Carlsbad, CA, USA) for 1 h30 min. Optical density (OD) was then read at the wavelengths of 570 and 600 nm in a spectrophotometer (SPECTRA Max 340 PC) to determine cell viability, expressed in percentage terms, used to calculate the IC_50_ values. These were determined by applying sigmoidal regression to the concentration-response curves using GraphPad Prism v5.0^30^ from at least three independent experiments performed in triplicates.

### Cytotoxicity assay

Human THP-1 cells were centrifuged at 300 × g for 10 min at 4 °C and resuspended (10^5^ cells/100 µL) in complete RPMI medium containing 100 nM phorbol 12-myristate 13-acetate (PMA), then distributed on 96-well plates. Cell cultures were incubated at 37 °C for 72 h to induce macrophage differentiation. The wells were then washed twice with saline, the complete RPMI medium (without PMA) was replenished and all cells were reincubated at 37 °C for an additional 48 h. Cultures were then treated with GA, 17-AAG or 17-DMAG in 12-step serial dilutions (1:2) using an initial concentration of 50 μM. After 72 h of treatment at 37 °C, cells were then incubated with 10% Alamar Blue^®^ for 22 h, after which plates were read at wavelengths of 570 and 600 nm in a spectrophotometer (SPECTRA Max 340 PC). The obtained data were used to calculate the percentage of cell viability and, subsequently, the CC_50_ value was determined from sigmoidal regression of the concentration-response curves using GraphPad Prism (version 5.0) from at least three independent experiments performed in triplicate. Cytotoxicity assays were also performed with human lung fibroblasts (MRC-5 cells). To this end, MRC-5 cells were centrifuged at 300 × g for 10 min at 4 °C and plated (2.5 × 10^4^ per well) in complete RPMI medium and left for 24 h. The cells were then treated with 17-AAG or 17-DMAG (initial concentrations: 400 µM and 50 μM, respectively) following the same protocol for THP-1 cells. After 72 h, Alamar blue® was added and the plates were read in a spectrophotometer. CC_50_ values were determined in the same manner as for THP-1 cells.

### Intracellular viability assay

To demonstrate the inhibitors’ activity against amastigotes of *L*. *amazonensis*, intracellular viability assays were performed using 17-DMAG. THP-1 cells (5 × 10^5^ per well) were plated in complete RPMI medium with PMA (100 nM) at 37 °C to promote differentiation into macrophages. After 72 h, cells were washed with saline and the medium (without PMA) was replenished. After 48 h in medium without PMA, the cells were washed again and then infected with *L*. *amazonensis* promastigotes in stationary phase (10:1) for 6 h. To remove non-internalized parasites, cells were washed twice with saline, the medium was replenished and concentrations of 6.25 nM, 12.5 nM, 25 nM, 50 nM, 100 nM, 150 nM, 200 nM, 250 nM, 300 nM and 400 nM (five replicates for each concentration) of 17-DMAG were added, followed by an incubation period of 72 h. Cells were then washed with saline, Schneider’s complete medium was added and cultures were kept at 24 °C. After four days, parasites were counted in a Neubauer chamber and the IC_50_ value was calculated for 17-DMAG.

### Statistical analysis

Graphing and statistical analysis of *in vitro* data was performed using the GraphPad Prism program (version 5.0). The Mann-Whitney test was used for comparisons between two groups with non-Gaussian distribution.

### *In silico* approach

The initial structure of GA was obtained from a co-crystallized complex of the NTD of human Hsp90 and GA (PDB code 1YET, available at the RCSB Protein Data Bank, http://www.rcsb.org). The structures of 17-AAG and 17-DMAG were constructed by performing the appropriate modifications to the obtained GA structure using the SPARTAN’08 program^31^. The Balloon program^32^ was used to generate conformer ensembles for each of the three macrocyclic Hsp90 inhibitors, GA, 17-AAG and 17-DMAG. The program settings used to create these new macrocyclic ring conformations are available as Supplementary Information (Supplementary Table S1). The optimization of these conformations was carried out using the MMFF (Merck Molecular Force Field) molecular mechanics method, available in the SPARTAN’08 program, using the internal default settings for convergence criteria.

The structure of the NTD of *L*. *amazonensis* Hsp83 was modeled using the SWISS-MODEL homology-modeling tool^33^ by inputting the *L*. *amazonensis* Hsp83 amino acid sequence available from NCBI (GenBank code AAA29250.1, available at National Center Biotechnology Information, http://www.ncbi.nlm.nih.gov). The 3 h80.1.A model was selected for use in this study, with 93.20% of sequence identity with the primary structure of the protein. This model has been constructed based on *L*. *major* Hsp83 structure as the template.

Docking analysis was carried out on each of the Hsp90 inhibitors and the NTD of human Hsp90 (PDB code 1YET), as well as modeled NTD of *L*. *amazonensis* Hsp83. All atoms within a radius of 15.0 Å around co-crystallized GA were considered to define the binding site location used for the analysis of the other two inhibitors. GOLD 5.2 software^34^ was used for docking calculations using the ChemPLP score function, which is an empirical energy-based scoring function that evaluates advanced protein-ligand docking by ranking the generated ligand binding poses. Using a conformational library, the following *L*. *amazonensis* Hsp83 residues were treated as flexible: Asn30, Ser33, Asn34, Asp37, Lys41, Asp76, Met81, Asn89, Leu90 and Phe121; for human Hsp90, the residues treated as flexible were Leu48, Asn51, Ser52, Asp93, Met98, Asp102, Asn106, Lys112, Thr152 and Thr184. The water molecules (HOH) within the binding site were considered in our docking calculations, allowing for the translation, spin and moving of these water molecules up to a distance of 2 Å away from the initial co-crystalized position. The BINANA^35^ program was then employed to analyze the molecular interactions resulting from docking using default settings. Finally, we used the PyMOL molecular visualization system to generate protein structure images^36^.

## Supplementary information

Supplementary Information
