# Supplementary material for: A docking-based structural analysis of geldanamycin-derived inhibitor binding to human or Leishmania Hsp90
Source: Sci Rep. 2019 Oct 14;9:14756. doi: 10.1038/s41598-019-51239-0 (PMC6791876; doi:10.1038/s41598-019-51239-0)
Supplement: Supplementary file 1 — Supplementary Information [file 41598_2019_51239_MOESM1_ESM.pdf]

A docking-based structural analysis of geldanamycin-derived inhibitor binding to human or *Leishmania* Hsp90

Luana Carneiro Palma[1], Luiz Felipe Gomes Rebello Ferreira[2], Antonio Luis de Oliveira Almeida Petersen[1], Beatriz Rocha Simões Dias[1], Juliana Perrone Bezerra de Menezes[1], Diogo Rodrigo de Magalhães Moreira[3], Marcelo Zaldini Hernandes[2] and Patricia Sampaio Tavares Veras[1, *]

[1]Gonçalo Moniz Institute, FIOCRUZ, Laboratory of Parasite – Host Interaction and Epidemiology, Salvador, 40296-710, Brazil

[2]Federal University of Pernambuco, Department of Pharmaceutical Sciences, Recife, 50670-901, Brazil

[3]Gonçalo Moniz Institute, FIOCRUZ, Laboratory of Tissue Engineering and Immunopharmacology, Salvador, 40296-710, Brazil

*patricia.veras@fiocruz.br

Supplementary Table S1.Settings used to create the 5 new conformations for the macrocyclicring of HSP90 inhibitors, using the program Balloon.

**Parameter**	**Value**
-c	-
--listAtomTypes	-
-k	-
-fullforce	-
--randomSeed	1000
-v	2
-i	500
--maxtime	10000000
--nGenerations	1000
--nicheRadius	1.5
-R	1.0
--maxPostprocessIter	500
--maxShapeIterations	500
--tournamentSize	20

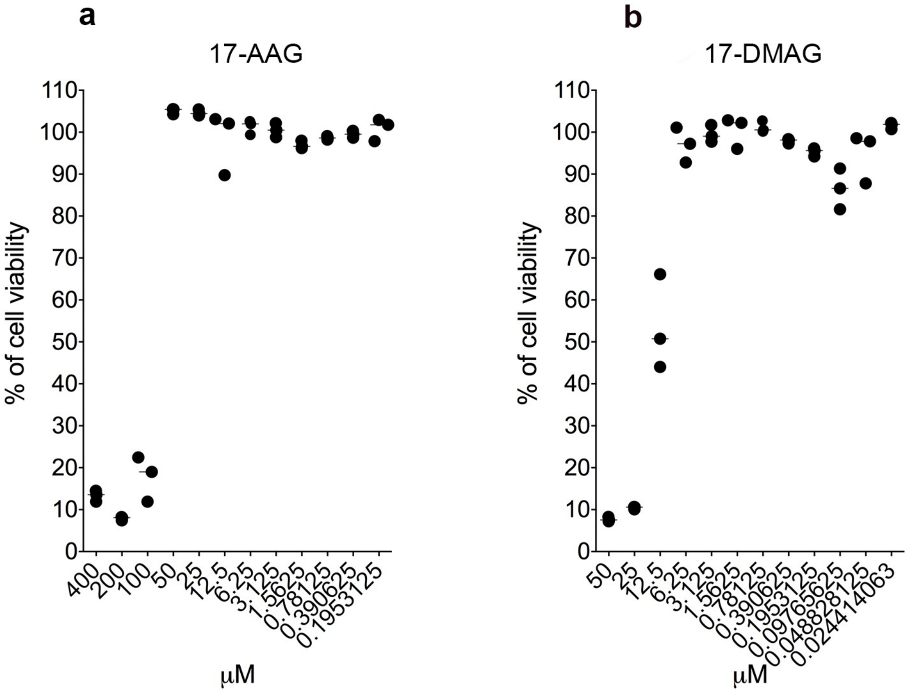

Supplementary Figure S1. Cell viability of MRC-5 cells treated with 17-AAG or 17-DMAG. MRC-5 cells were treated with 17-AAG (a) or 17-DMAG (b) in 12-step serial dilutions (1:2) (initial concentrations: 400 µM and 50 µM, respectively). After 72 h, Alamar blue® was added, and the plates were read in a spectrophotometer (570 and 600nm). Then, the obtained data were used to calculate the percentage of cell viability.

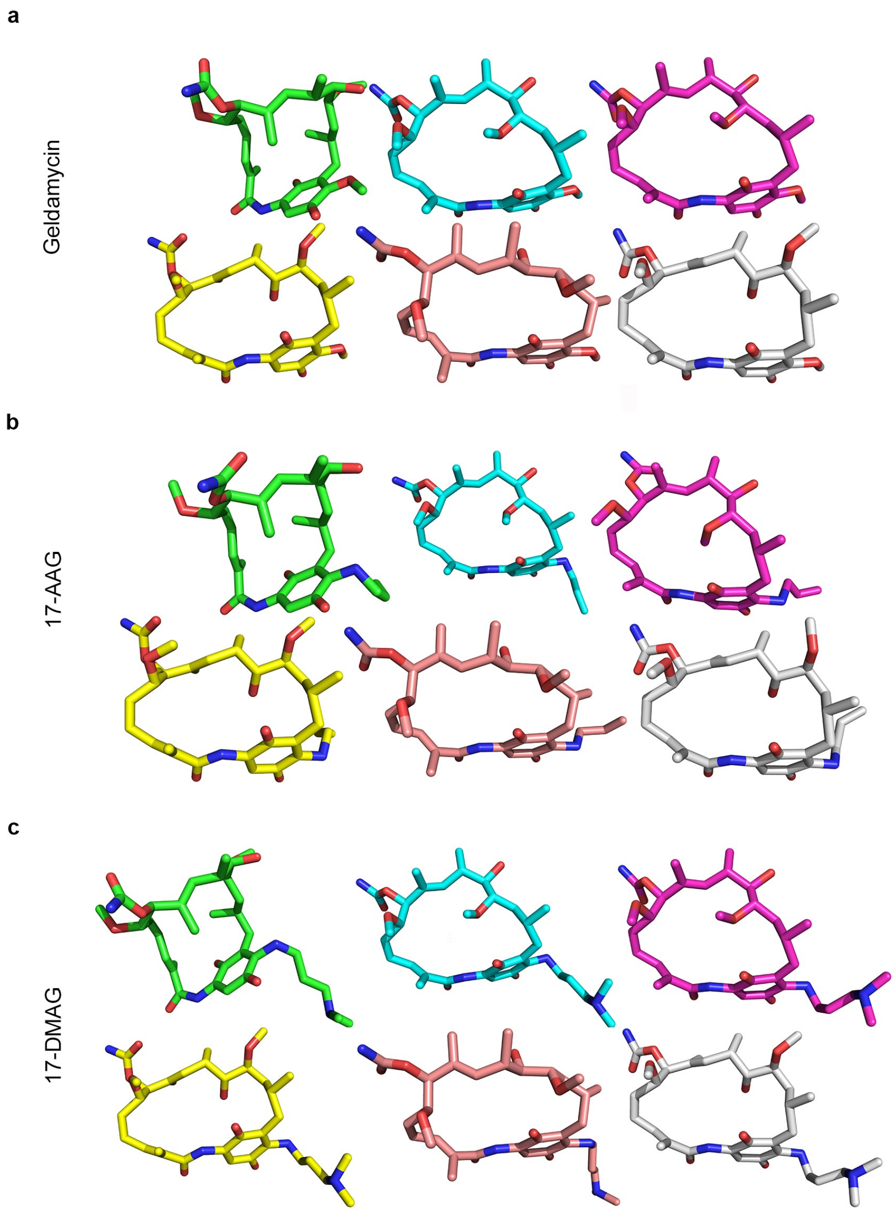

Supplementary Figure S2. Six conformations of HSP90 inhibitors used in this work.Conformations of geldanamycin (a), 17-AAG (b) and 17-DMAG (c) were generated using Balloon program. The macrocyclic ring of each six conformationsis identical among the threeHSP90 inhibitors.The only difference is in the substituent groups.
